# Potential Therapeutic Targeting of lncRNAs in Cholesterol Homeostasis

**DOI:** 10.3389/fcvm.2021.688546

**Published:** 2021-06-10

**Authors:** Wen-Chu Ye, Shi-Feng Huang, Lian-Jie Hou, Hai-Jiao Long, Kai Yin, Ching Yuan Hu, Guo-Jun Zhao

**Affiliations:** ^1^The Sixth Affiliated Hospital of Guangzhou Medical University, Qingyuan People's Hospital, Qingyuan, China; ^2^Xiangya Hospital, Central South University, Changsha, China; ^3^Guangxi Key Laboratory of Diabetic Systems Medicine, The Second Affiliated Hospital of Guilin Medical University, Guilin Medical University, Guilin, China; ^4^Department of Human Nutrition, Food and Animal Sciences, College of Tropical Agriculture and Human Resources, University of Hawaii at Manoa, Honolulu, HI, United States

**Keywords:** cholesterol homeostasis, lncRNAs, liver disease, lipid-related diseases, cardiovascular disease

## Abstract

Maintaining cholesterol homeostasis is essential for normal cellular and systemic functions. Long non-coding RNAs (lncRNAs) represent a mechanism to fine-tune numerous biological processes by controlling gene expression. LncRNAs have emerged as important regulators in cholesterol homeostasis. Dysregulation of lncRNAs expression is associated with lipid-related diseases, suggesting that manipulating the lncRNAs expression could be a promising therapeutic approach to ameliorate liver disease progression and cardiovascular disease (CVD). However, given the high-abundant lncRNAs and the poor genetic conservation between species, much work is required to elucidate the specific role of lncRNAs in regulating cholesterol homeostasis. In this review, we highlighted the latest advances in the pivotal role and mechanism of lncRNAs in regulating cholesterol homeostasis. These findings provide novel insights into the underlying mechanisms of lncRNAs in lipid-related diseases and may offer potential therapeutic targets for treating lipid-related diseases.

## Introduction

Cholesterol is a crucial organic molecule that exerts pleiotropic functions. Although its presence is crucial to the cell membranes' permeability and fluidity, excessive cholesterol in the bloodstream can be harmful. Therefore, maintaining cholesterol homeostasis is vital to normal cellular functioning. Cellular cholesterol maintains homeostasis by regulating cholesterol synthesis, cholesterol efflux, and cholesterol uptake from lipoprotein carriers (1, 2). The aberrant trafficking and cellular cholesterol homeostasis usually lead to various diseases, including obesity, diabetes, cardiovascular disease, and cancer (2, 3). Recent research suggest that disrupted cholesterol homeostasis can also cause various congenital diseases (4, 5). A growing body of evidence offers a close relationship between cholesterol homeostasis and acquired diseases, including cardiovascular disorders, liver diseases, and several types of cancer.

Long non-coding RNAs (lncRNAs), non-protein coding transcripts longer than 200 nucleotides (>200 bp) are important regulators of genome structure and gene expression. Recently, the contribution of lncRNAs in cholesterol homeostasis has just started to emerge (6, 7). Numerous studies demonstrated that through various regulatory mechanisms, lncRNAs regulate cell development and cell type-specific expression patterns. LncRNAs influenced cholesterol homeostasis and the progression and development of lipid-related diseases, including liver and cardiovascular disease (8, 9). With progress in next-generation sequencing technology, novel lncRNAs have been recognized and their diverse functions identified. However, the regulation by which many of the lncRNAs exhibit their functions is poorly understood. The low homology and conservation of lncRNAs across species pose a problem for developing lncRNA-based therapies. This review summarizes the latest insights on the roles of lncRNAs in cholesterol homeostasis and their potential implication for the treatment of lipid-related diseases.

## M^6^A-Modified lncRNAs Involved in Lipid Metabolism

N^6^-methyladenosine (m^6^A) modification, the most prevalent epigenetic methylated modification of lncRNAs in eukaryotes, plays a critical role in RNA splicing, export, stability and translation (10, 11). m^6^A modification is a dynamic and reversible process that is modulated by m^6^A regulators, including “writers” (methyltransferases), “readers” (signal transducers) and “erasers” (demethylases) (12). Recent research has revealed that m^6^A modification regulated lipid metabolism and lipid metabolic disorders in various lipid-related diseases, including liver disease, glioblastoma (GBM) and atherosclerosis (13, 14). For example, EGFR/SRC/ERK signaling-stabilized YTHDF2 (YTH N^6^-methyladenosine RNA binding protein 2) protein promoted cholesterol dysregulation and facilitated tumorigenesis of GBM cells (15); knockdown of methyltransferase like 14 (METTL14) limited cholesterol efflux (16). The resveratrol alleviated lipid metabolism disorder under HDF by increasing methyltransferase-like 3 (METTL3) expression and decreasing the level of YTHDF3 and m^6^A abundance (17). Thus, dysregulation of m^6^A modification is involved in the development of lipid-related disease (18).

Aberrant lncRNA expression is also strongly related to lipid metabolism disorders, and dysregulation of lncRNAs has been confirmed to play a vital role in the progression of liver disease and atherosclerosis (19, 20). For instance, liver-specific triglyceride regulator (lncLSTR), a liver-enriched lncRNA, increased triglyceride levels in a hyperlipidemia mouse model (21). RP11-728F11.4 promoted intracellular cholesterol accumulation and augmented atherosclerotic lesions (22). Knockdown of lncRNA MAARS (macrophage-associated atherosclerosis lncRNA sequence) inhibited atherosclerotic lesion formation in LDLR^−/−^ mice through tethering HuR in the nucleus (23).

Numerous studies have shown that m^6^A modification is closely related to the dysregulation of lncRNAs (24, 25). METTL3/YTHDF3 complex increased the stability and levels of MALAT1 and X inactive-specific transcript (XIST) (26, 27). MALAT1 and XIST were up-regulated in patients with cardiovascular disease and Stanford type A aortic dissection (TAAD) (28–30). MALAT1 facilitated hepatic lipid accumulation and promote atherosclerotic plaque formation (31, 32). XIST regulated the progression of atherosclerosis and TAAD (33). Thus, m^6^A modifications led to the lncRNA-dependent lipid-related disease development. Understanding how m^6^A modifications of lncRNAs are involved in lipid-related disease progression may help identify biomarkers that can act as useful therapeutic targets.

## Regulation of Cholesterol Homeostasis by lncRNAs

**Figure 1:**
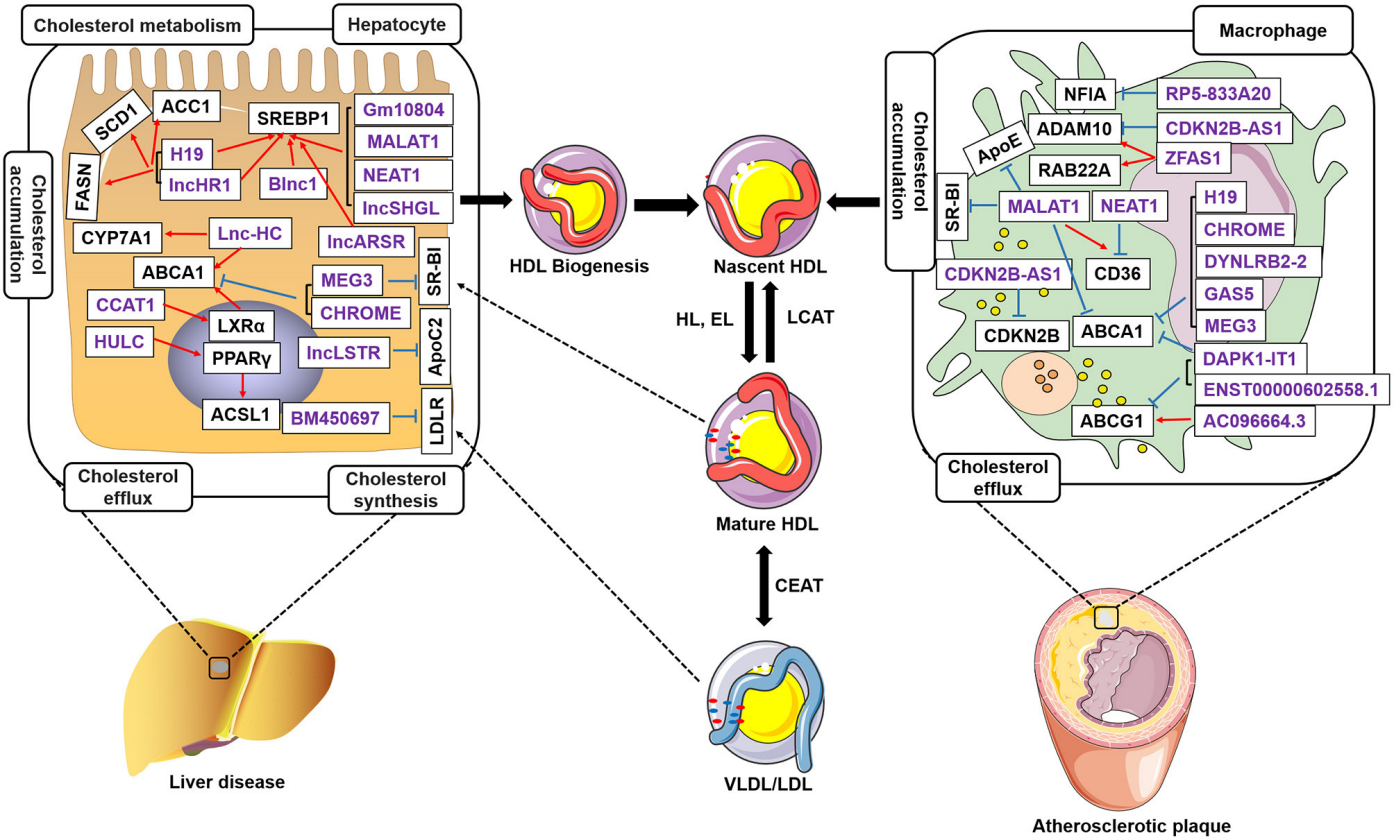
LncRNAs regulate cholesterol homeostasis in hepatocytes and macrophages. LncRNAs in the liver regulate cholesterol accumulation, cholesterol efflux, cholesterol biosynthesis, and cholesterol metabolism. LncRNAs control cholesterol accumulation and cholesterol efflux in atherosclerotic plaques. LncRNAs regulate ABCA1 expression and inhibit cholesterol efflux to lipid poor apoA-I, which initiates nascent high-density lipoproteins (HDLs). LncRNAs control SREBP1, ACC1, SCD1, FASN, CYP7A1, and ACSL1 expression in hepatocytes. Meantime, lncRNAs also regulate NFIA, ApoE, CDKN2B, RAB22A, CD36, ABCG1, and ADAM10 expression in the macrophages accumulated in atherosclerotic plaques. Free cholesterol in the nascent HDL is further esterified to cholesteryl ester by lecithin-cholesterol acyltransferase (LCAT), which results in the formation of mature HDL particles. Purple indicates lncRNAs. Black represents the target genes regulated by LncRNAs. Blue, inhibit; Red, promote.

**Figure 2:**
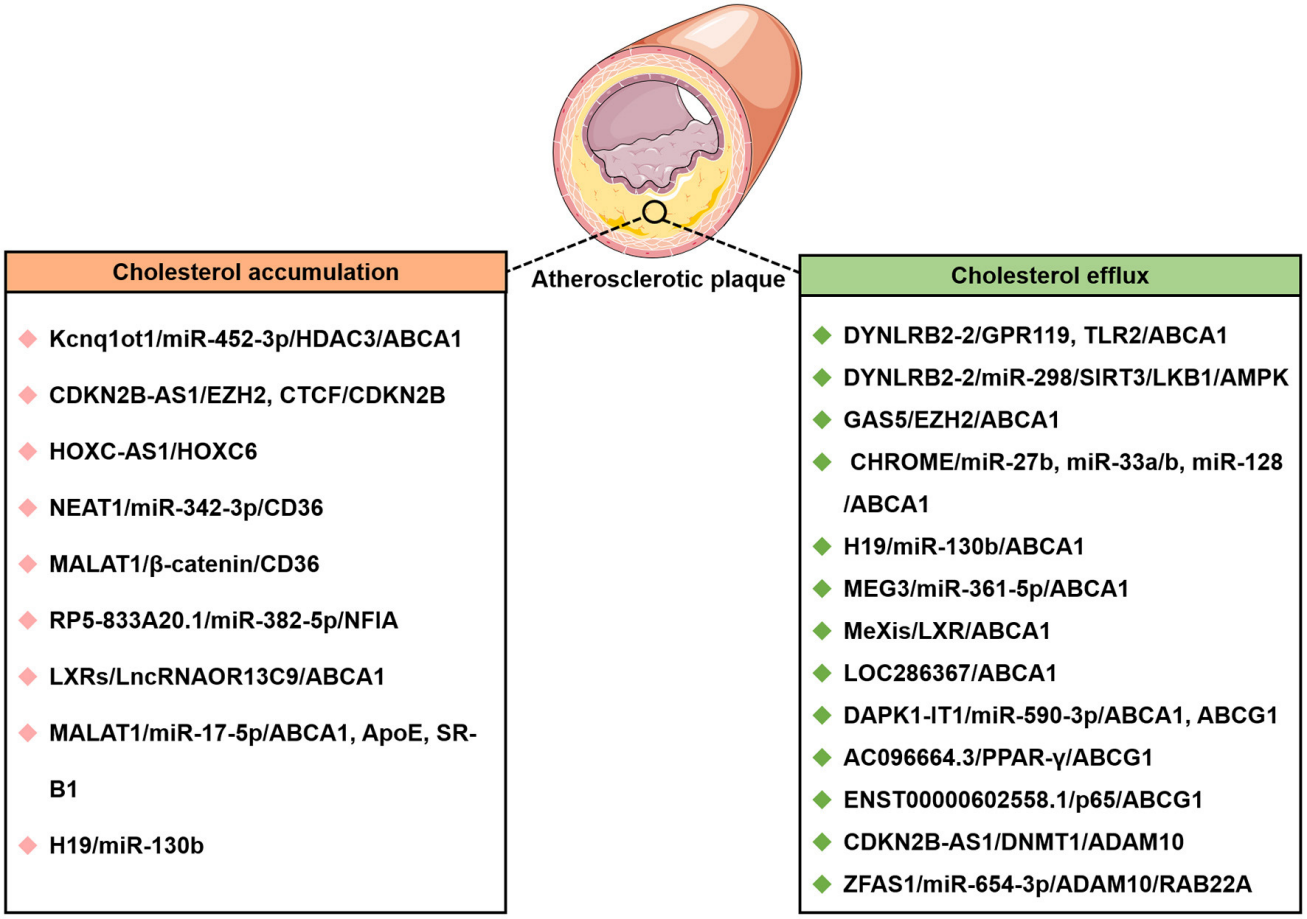
The lncRNAs affect cholesterol accumulation and cholesterol efflux from THP-1 macrophage-derived foam cells in atherosclerotic disease. CTCF, CCCTC-binding factor; DNMT1, DNA methyltransferase 1; EZH2, enhancer of zeste homolog 2; HOXC-AS1, lncRNA HOXC cluster antisense RNA 1; Kcnq1ot1, Kcnq1 overlapping transcript 1; LncRNA MAARS, Macrophage-Associated Atherosclerosis lncRNA Sequence; NFIA, nuclear factor IA.

**Figure 3:**
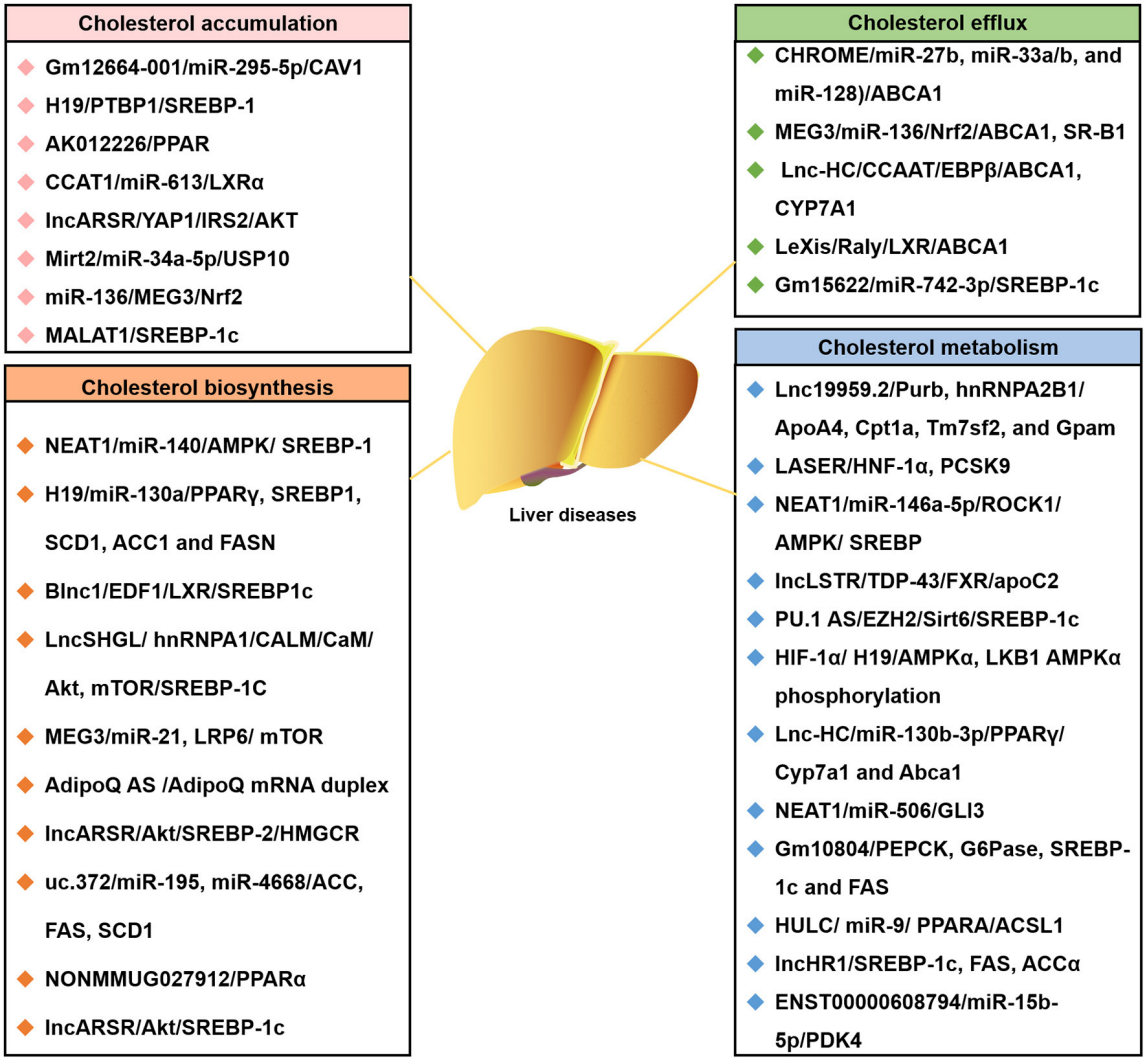
The lncRNA-associated ceRNA networks affect the four common cholesterol transport models of liver diseases. Representative lncRNA-ceRNA networks are listed. They highlighted the involvement of lncRNA-ceRNA networks in four common cholesterol transport models of liver diseases: cholesterol accumulation, cholesterol efflux, cholesterol biosynthesis, and cholesterol metabolism. ACCα, Acetyl-CoA carboxylase α; CaM, calmodulin; FAS, fatty acid synthase; G6Pase, glucose-6-phosphatase; hnRNPA1, heterogeneous nuclear ribonucleoprotein A1; LncLSTR, liver-specific triglyceride regulator; LncSHGL, hepatic gluconeogenesis, and lipogenesis; Mirt2, long non-coding RNA myocardial infarction associated transcript 2; PEPCK, phosphoenolpyruvate carboxykinase.

In the last few years, evidence has emerged that lncRNAs play a key role in cholesterol accumulation, cholesterol efflux, cholesterol metabolism, and cholesterol biosynthesis (Table 1 and Figure 1), which were implicated in lipid-related diseases, including liver diseases and cardiovascular diseases (52, 53). Moreover, several lncRNAs participated in the regulation of cholesterol homeostasis in the aorta/macrophages (Figure 2). Some lncRNAs played crucial roles in cholesterol homeostasis in the liver (Figure 3). This review provides a comprehensive insight into the current knowledge regarding lncRNAs involved in regulating cholesterol homeostasis, which identifies potentially useful therapeutic targets for cholesterol modulation.

**Table 1:** Summary of lncRNAs diseases with disturbed cholesterol homeostasis.

**lncRNAs**	**Target genes**	**Function**	**Diseases**	**References**
**Cholesterol efflux**				
CDKN2B-AS1	ADAM10	cholesterol efflux (+)	Atherosclerosis	(34)
CHROME	miR-27b, miR-33a/b and miR-128	Cholesterol efflux (+)	CAD	(35)
ENST00000602558.1	p65	Cholesterol efflux (+)	CAD	(36)
MALAT1	SREBP-1c	Cholesterol efflux (+)	Hepatic steatosis and insulin resistance	(31)
**Lipid accumulation**				
GAS5	miR-135a	Lipid accumulation (+)	Atherosclerosis	(37)
MEG3	miR-21	Lipid accumulation (+)	NAFLD	(38)
H19	MLXIPL and mTORC1	Lipid accumulation (+)	NAFLD	(39)
MALAT1	miR-17-5p	Lipid accumulation (+)	Atherosclerosis	(40)
HOXC-AS1	HOXC6	Lipid accumulation (+)	Atherosclerosis	(41)
HOTAIR	NF-κB	Lipid accumulation (+)	Atherosclerosis	(42)
NEAT1	miR-146a-5p	Lipid accumulation (+)	NAFLD	(43)
**Lipid metabolism**				
Lnc-HC	miR-130b-3p	Lipid metabolism (+)	NAFLD	(44)
ENST00000416361	SREBP1, SREBP2	Lipid metabolism (+)	CAD	(45)
LeXis	RALY	Lipid metabolism (−)	–	(46)
**Lipid desaturation**				
LINC01138	PRMT5	Lipid desaturation (+)	Kidney malignancy	(47)
**HDL-C and LDL-C level**				
SNHG17	–	HDL-C level (−)	Type 2 diabetes mellitus	(48)
TTC28-AS1	–	LDL-C level (+)	Type 2 diabetes mellitus	(48)
MIAT	HIF1α, ALKBH1	LDL-C level (+)	Atherosclerosis	(49)
LINC00958	miR-382-5p	HDL-C (+) and LDL-C level (−)	Atherosclerosis	(50)
**Hepatic lipogenesis**				
LINC00958	miR-3619-5p	Hepatic lipogenesis (+)	HCC	(51)
Blnc1	SREBP1c	Hepatic lipogenesis	Obesity and NAFLD	(20)

### MIAT

The lncRNA myocardial infarction-associated transcript (MIAT), as a hypoxia-response gene, is located in chromosome 22q12.1 region. MIAT was markedly elevated in the serum of patients with symptoms of vulnerable atherosclerotic plaque (54). MIAT increased the blood lipids levels, promoted atherosclerotic plaque formation, increased the lipid content, and decreased the collagen content of atherosclerotic plaques in apoE^−/−^ mice (55). Silencing of MIAT attenuated atherosclerosis progression in an advanced atherosclerosis mouse model (54). However, MIAT overexpression aggravated the atherosclerotic damage in apoE^−/−^ mice (55). MIAT facilitated angiogenesis and the expression of inflammatory factors (IL-1β, IL-6, and TNF-a) by activating the PI3K/Akt pathway. MIAT was the target gene of m^6^A modification. m^6^A level was reduced with enlarged carotid plaque size and thickness in 207 patients with atherosclerosis compared with 142 healthy people (49). ox-LDL-induced AlkB homolog 1 (ALKBH1) and m6A demethylation further promoted MIAT activity with the hypoxia-inducible factor 1α (HIF1α) motif (−1,940/+166-Luc plasmids) but not with deletion (56). Deficiency of ALKBH1 or HIF1α by siRNA transfection strongly upregulated MIAT expression and the m^6^A levels *in vitro* (49). Therefore, MIAT may provide a novel target for the treatment of atherosclerotic disease.

### LINC00958

Long intergenic non-protein coding RNA 958 (LINC00958), a lipogenesis-related lncRNA, is located on chromosome 11p15.3 regions. LINC00958 was upregulated in hepatocellular carcinoma (HCC) cells, especially in those with moderate/low differentiation, TNM III/IV stage, and microvascular invasion. LINC00958 knockdown in HCC cells decreased cellular cholesterol and triglyceride levels, whereas LINC00958 overexpression increased cholesterol and triglyceride levels (51). METTL3-mediated m^6^A modification upregulated LINC00958 expression by stabilizing its RNA transcript and increased lipogenesis to promote HCC progression (51). LINC00958 upregulated hepatoma-derived growth factor (HDGF) expression by sponged miR-3619-5p (51). HDGF facilitated the expression of lipogenic genes, which promoted *de novo* lipogenesis and tumorigenesis. Thereby, LINC00958 augmented HCC lipogenesis and progression, implying that LINC00958 provided a novel perspective for targeted therapy of HCC.

### H19

The H19 gene belongs to the H19-Igf2 locus, is located in an imprinted region of chromosome 11p15.5 near the insulin-like growth factor 2 (IGF2) gene in humans. Compared with normal healthy people, the expression of H19 was higher in the blood of patients with atherosclerosis (57), suggesting that H19 may be involved in atherosclerosis progression. In apoE-/- mice, overexpression of H19 aggravated atherosclerosis progression (58); however, silencing of H19 protected against atherosclerosis (59). Recently, H19 was reported to modulate hepatic metabolic homeostasis in non-alcoholic fatty liver disease (NAFLD). H19 promoted lipogenesis by directly inhibiting miR-130a expression in hepatocytes (60). Meanwhile, miR-130a inhibited lipid accumulation by directly down-regulating peroxisome proliferator-activated receptor γ (PPARγ) expression (60, 61). PPARγ was shown to promote cholesterol efflux by regulating ABCA1 and ABCG1 in plaque *in vivo* and phagocytes *in vitro*, which could be blocked by PPARγ siRNA (61). Overexpression of H19 in hepatocytes also promoted lipid accumulation and upregulated the expression of multiple genes involved in lipid synthesis, storage, and breakdown, while deficiency of H19 resulted in decreased lipid accumulation in hepatocytes (39). Therefore, H19 may become a new target for the therapy of lipid-related diseases, such as liver disease and cardiovascular disease.

### GAS5

LncRNA growth arrest-specific 5 (GAS5), located on human chromosome 1q25.1, plays a crucial role in atherosclerosis's pathogenesis. GAS5 was significantly increased in atherosclerosis patients' plaque than in normal people (62).

Overexpression of GAS5 increased lipid accumulation via inhibiting enhancer of zeste homolog 2 (EZH2)-mediated ABCA1 expression by histone methylation in THP-1 macrophage. In contrast, GAS5 knockdown promoted reverse-transportation of cholesterol and inhibited lipid accumulation by upregulating the expression of ABCA1 (63). GAS5 overexpression in apoE^**−/−**^ mice with atherosclerosis also increased total cholesterol (TC), free cholesterol (FC), cholesterol ester (CE), low-density lipoprotein (LDL) levels, aortic plaque, and lipid accumulation; however, silencing of GAS5 prevented the progression of atherosclerosis (63). Previous studies have shown that GAS5 silencing repressed atherosclerosis's malignant progression (37). Thus, targeting GAS5 might be a promising way for atherosclerosis therapy.

### CHROME

Cholesterol-induced regulator of metabolism RNA (CHROME), also known as PRKRA-AS1, is located in a locus on human chromosome 2q31.2, regulates cellular and systemic cholesterol homeostasis. Analysis of blood and tissue samples from healthy individuals and coronary artery disease (CAD) patients revealed that CHROME is upregulated in the plasma and atherosclerotic plaques of patients with the atherosclerotic disease (35). Using gain- and loss-of-function approaches, CHROME promoted cholesterol efflux and HDL biogenesis in the liver and macrophages via inhibiting the actions of functionally related miRNAs, such as miR-27b, miR-33a/b, and miR-128. Conversely, CHROME knockdown inhibited ABCA1 expression in human hepatocytes and macrophages, which blocks cholesterol efflux and the formation of nascent high-density lipoprotein (HDL) (35). Therefore, CHROME may be a clinical biomarker for treating cholesterol-related diseases.

### MEG3

Maternally expressed gene 3 (MEG3) is a lncRNA located in a locus on chromosome 14q32.2 thought to be associated with human lipid metabolic disorders. A study recently demonstrated that the expression of MEG3 was reduced in serum samples from patients with atherosclerosis (64). MEG3 deficiency remarkably abolished hepatic TG accumulation in HFD mice and ob/ob mice (65, 66). MEG3 alleviated NAFLD after high-content hydrogen water treatment in a mouse model (65). MEG3 expression is negatively correlated with lipogenesis-related genes, including sterol regulatory element-binding protein-1 (SREBP-1), LXRα, Carbohydrate response element-binding protein (ChREBP), Stearyl-coenzyme A desaturase 1 (SCD1), acetyl-CoA carboxylase 1 (ACC1), and fatty acid synthase (FAS), in NAFLD mice (38). MEG3 overexpression significantly inhibited the expression levels of lipogenesis-related genes and lowered FFA-induced lipid accumulation in HepG2 cells. Bioinformatic analysis and mechanistic studies illustrated that MEG3 competitively bound to the miR-21 with LRP6, followed by the inhibition of the mTOR pathway and inhibited hepatic lipogenesis (38). Therefore, the targeted suppression of MEG3 may serve as a potential therapy for lipid-related diseases.

### LeXis

LeXis is a lipid-responsive lncRNA, highly expressed in the hepatic tissue and robustly induced by Western diet (high in fat and cholesterol) and pharmacologic liver X receptors (LXRs) activation (46). Hepatic overexpression of LeXis in mice decreased plasma cholesterol, whereas LeXis knockout mice had the opposite phenotype of increased serum cholesterol level and upregulated cholesterol biosynthetic gene expression (67). Raising or lowering LeXis levels in the liver and plasma affected cholesterol biosynthesis and altered the cholesterol levels by LXRs activation. LXRs are transcriptional regulators of cholesterol homeostasis. Under conditions of excess cholesterol, LXR activation-induced apoE, ABCA1, and ABCG1 expression, which was involved in cholesterol efflux, facilitated cholesterol esterification and inhibited cholesterol uptake (46, 68). Overexpression of LXRs significantly promoted cholesterol efflux via the upregulation of ABCA1 and ABCG1 (69); conversely, shRNA-mediated knockdown suppressed ABCA1 and ABCG1 expression and promoted intracellular cholesterol accumulation (70). Taken together, LeXis has important implications in developing novel therapeutic strategies for treating lipid-related diseases.

### CDKN2B-AS1

CDKN2B-AS1, also known as ANRIL, is located within the CDKN2B-CDKN2A gene cluster at chromosome 9p21 in humans. Prior studies have demonstrated that it was expressed significantly higher in hypertension patients than in healthy controls and was particularly associated with cardiovascular disease (71). Transcript variants of CDKN2B-AS1 have also been shown to play critical regulatory roles in various diseases, including malignant tumors, atherosclerosis, hypertension, and diabetes (72–75). CDKN2B-AS1 promoted cholesterol efflux by inhibiting A disintegrin and metalloprotease 10 (ADAM10) expression in atherosclerosis (34). Overexpression of ADAM10 facilitated the intracellular accumulation of cholesterol, while knockdown of ADAM10 promoted cholesterol efflux. Hence, CDKN2B-AS1 may serve as a biomarker for atherosclerosis.

### LASER

A novel lncRNA, lipid Associated Single nucleotide polymorphism gEne Region (LASER), is located near SNP rs486394 in chromosome 11q12 region. Clinical studies previously revealed that LASER expression is positively associated with cholesterol levels. LASER is highly expressed in both hepatocytes and peripheral mononuclear cells (PBMCs). siRNAs mediated knockdown of LASER improved intracellular cholesterol levels and affected the cholesterol metabolism genes expression at both protein and mRNA levels by inhibiting proprotein convertase subtilisin/kexin 9 (PCSK9) expression (76). PCSK9, a major determinant of cholesterol homeostasis, is mainly secreted from the liver and enhances circulating low-density lipoprotein cholesterol (LDL-C) concentrations in circulating blood (77). Thus, targeting LASER therapy may be a practical approach to ameliorate cholesterol levels in clinics.

### HOXC-AS1

LncRNA HOXC cluster antisense RNA 1 (HOXC-AS1) is located in chromosome 12q13.13 regions and has two exons. By performing microarray analysis and RT-PCR, the expression levels of HOXC-AS1 and homeobox C6 (HOXC6) were both downregulated in human atherosclerotic plaques when compared to normal intima tissues (41). Lentivirus-mediated overexpression of HOXC-AS1 suppressed ox-LDL-induced cholesterol accumulation by promoting HOXC6 expression in THP-1 macrophages (41). Numerous studies have reported that HOX gene networks are involved in human adipogenesis; notably, HOXC6 inhibited intracellular lipid accumulation (78). Thus, HOXC-AS1 could be a promising therapeutic target in preventing atherosclerosis.

### lncARSR

lncRNA regulator of Akt signaling associated with HCC and RCC (LncARSR) is located in chromosome 9q21.31 regions. The lncARSR expression level was increased both in patients with hypercholesterolemia and high-cholesterol diet-fed mice (79). Adenoviruses-mediated overexpression of lncARSR in mice contributed to elevated lipid levels in both serum and liver fragments. However, knockdown of lncARSR in mice fed with a high cholesterol diet exhibited a marked reduction in plasma lipid levels than control mice (79). Moreover, lncARSR overexpression facilitated HMG-CoA reductase (HMGCR) expression and the rate-limiting enzyme of cholesterol synthesis, accompanied by the augment of hepatic *de novo* cholesterol synthesis rate. Mechanistically, lncARSR promoted the expression of SREBP-2, which regulated the expression of cholesterol-related genes, such as HMGCR and LDLR (80). Hence, lncARSR promoted hepatic cholesterol biosynthesis and implied that lncARSR might serve as a therapeutic target for cholesterol homeostasis disorder.

### ENST00000602558.1

ENST00000602558.1 is located on a CAD, triglyceride (TG), and HDL susceptibility region (chr12q24.31) (81, 82). Li et al. performed a transcriptome-wide overview of aberrantly expressed lncRNAs in CAD patients, ENST00000444488.1 was identified as a novel lncRNA biomarker for diagnosing CAD (83). Overexpression of ENST00000602558.1 downregulated ABCG1 expression and exacerbated lipid accumulation in VSMCs, while knockdown of ENST00000602558.1 upregulated ABCG1 expression and decreased lipid accumulation (36). Thus, ENST00000602558.1 may be a novel biomarker for diagnosing atherosclerosis.

### LOC286367

LOC286367 is located in the chromosome 9q31.1 region. By performing bioinformatic analysis of lncRNAs and mRNA differentially expressed in THP-1 macrophages, Ma et al. proposed that LOC286367 and ABCA1 were located on the same chromosome with opposite transcription directions (84). Overexpression of LOC286367 inhibited ABCA1 expression, which resulted in intracellular lipid accumulation (84). ABCA1 overexpression in C57BL/6 mice resulted in an anti-atherogenic profile with reduced plasma cholesterol, free cholesterol, cholesteryl ester, and non-high-density lipoprotein cholesterol (HDL-C) levels, but with increased HDL-C, apoA-I, and apoE levels (85). However, ABCA1 knockout mice displayed increased atherosclerosis compared to control mice (86). Hence, targeting LOC286367 might bring significant benefits to the clinical outcome of atherosclerotic cardiovascular diseases.

### RP5-833A20.1

RP5-833A20.1 is located in intron 2 of the nuclear factor IA (NFIA) gene. RP5-833A20.1 expression was upregulated, whereas NFIA expression was downregulated in human acute monocytic leukemia macrophage-derived foam cells using microarray analysis (50). RP5-833A20.1 regulated cholesterol homeostasis by NFIA. Lentivirus-mediated NFIA overexpression increased HDL-C circulation, decreased LDL-C cholesterol, and very-low-density lipoprotein cholesterol (VLDL-C) circulation (50), which resulted in the regression of atherosclerosis in apoE^**−/−**^ mice. Thus, RP5-833A20.1 may represent a therapeutic target to ameliorate lipid-related diseases.

## Therapeutic Use of lncRNAs in Diseases

During the last decades, developments in genome-wide analyses have confirmed that almost all human genomes are transcribed with lncRNAs. Many lncRNAs have been known to be functional in mammals and are involved in various physiological and pathophysiological processes by epigenetics and transcriptional or post-transcriptional regulatory mechanisms (87, 88). Recently, many studies have demonstrated that lncRNAs are involved in the pathophysiology of various pathological conditions, including cancers (89), autoimmune diseases (90), and neurological disorders (91) and cardiovascular diseases (92).

Previous studies have demonstrated novel lncRNA biomarkers and identify therapeutic lncRNA targets (93, 94). A novel lnc030 was highly expressed in breast cancer (95). Inhibition of lnc030 expression by lentivirus-mediated short hairpins RNAs (shRNAs) reported markedly impaired colony formation. It also inhibited breast cancer initiation and progression, whereas ectopic lnc030 overexpression significantly increased colony formation and promoted initiation and progression of breast cancer (95). These results demonstrated that lnc030 could act as a therapeutic target and biomarker in breast cancer. Similarly, lncRNA PVT1 was verified to function as a tumor promoter in gastric cancer. It is reported that PVT1 was highly expressed in gastric cancer (GC) tissues, and high PVT1 level was correlated with tumor stage, lymph node metastasis, and poor prognosis (96). Overexpression of PVT1 greatly promoted the GC cell epithelial-to-mesenchymal transition (EMT) process and tumor metastasis *in vitro* and *in vivo* (96). These findings indicated that PVT1 has an important implication for future therapy of the GC. A similar vector has already been demonstrated as effective in animal studies for thyroid cancer therapy (97). Other circulating lncRNAs also have been verified as biomarkers in the diagnosis and prognosis of many diseases. For example, prostate-specific lncRNA prostate cancer antigen 3 (PCA3) levels have been suggested as a diagnostic biomarker of prostate cancer (98). Other lncRNAs used as biomarkers include circulating plasma H19 for gastric cancer (99), HULC in hepatocellular carcinoma (100), circulating lncRNA in SNHG11 colorectal cancer (101), circulating exosomal lncRNA-GC1 in gastric cancer (102), and HOTAIR in various cancers, including breast, colorectal, liver, gastric, lung, and thyroid (103–107).

Other than cancers, lncRNAs also have been investigated as promising biomarkers for atherosclerotic disease (108). CoroMarker was highly expressed in circulating peripheral blood monocytes (PBMCs) and plasma from patients with coronary artery disease (CAD) (109). CoroMarker acts as a candidate biomarker for CAD with an AUC of 0.920 and a 95% confidence interval of 0.892–0.947, and it could successfully distinguish CAD out of patients (109). CoroMarker is stable, sensitive, and mainly in the extracellular vesicle, probably from monocytes (110). LIPCAR has been identified from the plasma RNA from patients with myocardial infarction (111). LIPCAR is consistently detectable in the plasma and significantly increases in patients with myocardial infarction during later stages and ischemic and non-ischemic heart failure. Importantly, higher LIPCAR levels identified patients developing cardiac remodeling and were also reported to be an independent biomarker of future cardiovascular deaths (111). Another study compared the expression of lncRNAs in the peripheral blood cells between healthy and myocardial infarction patients. It demonstrated that in the cardiac hypertrophy-associated transcript (CHAST), MALAT1 was significantly upregulated in myocardial infarction patients (112–114). These findings provided a promising therapeutic strategy for treating atherosclerotic diseases and shed light on the clinical implication of lncRNA-associated ceRNA mechanisms in atherosclerotic disease deterioration.

## Clinical Application of lncRNAs in Lipid-Related Disease

lncRNAs may serve as biomarkers in human disease for diagnosis, prognosis, and therapy. This possibility is supported by preclinical studies and human studies that represent some of their characteristics. The characteristics of lncRNAs, including high tissue-specificity, disease specificity, cell-type specificity, and relative ease in detection methods, make them suitable for patients with diseases. Meantime, these data also suggested that lncRNAs are superior to proteins in terms of potential, targeted-related toxic effects. Moreover, lack of translation, rapid turnover and low expression levels may promote faster effects with lower doses.

Oligonucleotide therapeutics such as specific antisense oligonucleotides (ASOs), small interfering RNA technology, or small molecule inhibitors can also be used in treating a variety of diseases (Table 2), including cancer, infectious diseases, atherosclerosis, liver, and kidney disease (72, 129, 130). The most advanced therapeutic lncRNAs targeting currently are based on the use of ASOs. These molecules are short single-stranded DNA that could be synthesized based on unique RNA sequence and accessibility. Importantly, ASOs represent a valuable approach to antagonize lncRNAs, leading to premature transcription termination and inhibited lncRNA expression (131, 132). ASOs silencing of the expression of disease-related genes is a novel therapeutic approach. For example, LINC02273-targeting ASO significantly inhibited breast cancer metastasis *in vivo* (115). However, treatments using ASOs are limited in the clinic, mainly because of *in vivo* toxicity and the lack of a system for delivering ASOs to cells and tissues. To improve their pharmacological properties, ASOs are modified to improve potency, efficacy, stability (133), thereby increasing resistance to nucleases degradation and decreasing non-specific immune-stimulating activity. Several mRNA-therapeutic ASOs have already been approved by the Food and Drug Administration (FDA), and the European Medicines Agency and several others are in late-stage clinical development (134, 135). Some ASOs targeting lncRNA-dependent lipid-related disease are under development and protected by patents.

**Table 2:** Therapeutic aspects of lncRNAs in various diseases with oligonucleotide therapeutics.

**LncRNAs**	**Oligonucleotide therapeutics**	**Function**	**Diseases**	**References**
LINC02273	ASOs	Inhibit breast cancer metastasis	Breast cancer	(115)
AC104041.1	ASOs	Induced antitumor activity	Head and neck squamous cell carcinomas	(116)
MALAT1	ASOs	Inhibited tumor growth and metastasis	Breast cancer and Lung cancer	(117, 118)
ASMER-1/2	ASOs	Regulated adipogenesis, lipid mobilization and adiponectin secretion.	Obesity and/or the insulin resistant state.	(119)
**Small molecule inhibitors**				
GAS5	NP-C86	Increased GAS5 levels and glucose uptake in diabetic adipocytes	Diabetic	(120)
MIR155HG	NSC141562	Inhibition of glioma cell proliferation, migration, and invasion	Glioma	(121)
HULC	YK-4-279	Inhibition of Ewing sarcoma cell growth	Ewing sarcomas	(122)
HOTAIR	AC1Q3QWB	Blocked PRC2 recruitment and increased tumor suppressors expression	Breast cancer and glioblastoma	(123)
**CRISPR/Cas systems**				
PVT1	CRISPR interference	Enhanced breast cancer cell competition and growth	Breast cancer	(124)
UCA1	Knockout	Inhibition of tumorigenesis	Bladder cell carcinoma	(125)
LINC01116	Knockout	Decreased the ability of the prostate cancer cells	Prostate cancer	(126)
Lnc-PANDAR	Knockout	Inhibition of malignant features and tumor growth	Gastric cancer	(127)
MANTIS	Knockout	Inhibition of endothelial angiogenic function	Idiopathic pulmonary arterial hypertension	(128)
Blnc1	Knockout	Inhibition of hepatic steatosis and insulin resistance	Obesity and NAFLD	(20)

Small molecule inhibitors have been used for lncRNAs targeted therapies (136, 137). Obtaining small molecule inhibitors that bind lncRNAs with high affinity and specificity requires the identification of lncRNA motifs. This level of structural knowledge is available only for limited lncRNAs, suggesting that lncRNAs can form multiple functional domains involved in various molecular interactions to fight the disease (138, 139). Small molecule inhibitors can hamper interactions between lncRNAs and their associated protein partners, which could be desirable from a therapeutic perspective for disease. Recent studies have reported that lncRNA-protein interactions such as H19-EZH2 (140), HOTAIR-polycomb repressive complex 2 (PRC2) (141), and ANRIL-chromobox 7 (CBX7) (142) have become potential targets. Small molecule inhibitor, AC1Q3QWB, hampered the interaction between HOTAIR-EZH2 interaction in various cancer cell lines and orthotopic breast cancer models (123). In addition, synthetic functional molecules that mimic the lncRNAs' structure and binding properties may compete with lncRNA for protein binding, and thereby interfering with its function. These approaches will become more practical as the structural and binding properties of lncRNAs become better understood.

Clustered regularly interspaced short palindromic repeats (CRISPR) and CRISPR-associated proteins (Cas) (CRISPR/Cas) systems are the most versatile and promising within the past decade for modulation of lncRNAs (143). The CRISPR/Cas systems include CRISPR/Cas9, CRISPR interference (CRISPRi) (144) or CRISPR activator system (CRISPRa) (145), which were used for editing lncRNA-encoding genes. These tools ensure relatively rapid knockout, knockdown or overexpression of lncRNAs *in vivo* and *in vitro*. It has recently been widely used for research applications of a single lncRNA locus. It is increasingly applied to thousands of loci for high throughput functional screens in various experimental settings (146). However, because of their lack of protein-coding capacity, targeting lncRNAs *in vivo* using CRISPR/Cas systems is more difficult than targeting mRNA-coding genes. Therefore, we speculate that the therapeutic application of CRISPR/Cas systems at lncRNA loci will lag behind that of mRNA-coding genes.

Therapies based on the ASO and CRISPR/Cas systems have shown prominently good and long-lasting results, and are moving toward a portfolio of preventive treatments at the lipid levels. However, it should be noted that maintaining cholesterol homeostasis is a complicated process, and novel promising therapies for interventions affecting lipid metabolism are being discovered. For example, LASER has been implicated to participate in cholesterol homeostasis in hepatocytes and PBMCs (76). Likely, there are other yet unidentified lncRNAs and other RNA species that are also involved in this regulation.

## Conclusions and Future Directions

The importance of cholesterol homeostasis function is underscored by the diverse regulatory pathways that maintain cellular cholesterol levels within a narrow range. Cellular cholesterol deficiency and accumulation, hallmarks of some lipid-related diseases involving the liver and angiogenesis, highlight the importance of maintaining cholesterol homeostasis in various cell lines. In humans, cholesterol homeostasis is maintained by multiple feedback and compensatory mechanisms. LncRNAs have been involved in regulating cholesterol homeostasis, and several studies focused on lncRNAs regulated by cholesterol. These lncRNAs function in normal metabolism and cholesterol homeostasis as well as in the progression of lipid-related diseases. In this review, the discovery of lncRNAs has provided novel and sensitive biomarkers and therapeutic targets for patients with lipid-related diseases. Dysregulation in lncRNA expression could also be a cause of lipid-related diseases. Further, a lack of specific, secure, and effective delivery systems limit lncRNAs' use in treating lipid-related diseases. Future studies should focus on tissue-specific interference or overexpression of lncRNAs to achieve the targeted therapy of patients with lipid-related diseases.

Most lncRNAs frequently display tissue and disease-specific expression patterns and less conserved than protein-coding genes (147). Due to unique features, lncRNAs may be superior therapeutic targets than existing protein-coding genes for various disease diagnoses and prognoses. Additionally, lncRNAs act as biologically functional molecules, and their expression may be better biomarker candidates for various disease states (148). However, the function of most lncRNAs is still unknown, their role in physiology, development, and disease. Their effective use as therapeutic targets requires an enormous number of studies. Investigations are required to check the pharmacokinetics and toxicity of lncRNAs. It is also critical to further explore these regulatory RNAs' novel biologic characteristics and provide potential novel treatment options. However, lncRNA holds great therapeutic promise for potential intervention. Because of its tissue and disease-specific expression, lncRNA has great therapeutic interventions as a biomarker and an important therapeutic target for treating diseases. Considerable research is currently investigating the biological function of these lncRNAs for diagnostic, prognostic, and therapeutic. In this field, the research is increasing exponentially over the next decade will teach us more about the diagnostic, prognostic, and therapeutic of these lncRNAs.

## Author Contributions

G-JZ, CH, and W-CY designed and outlined the article. W-CY, L-JH, G-JZ, and CH conducted the literature article. W-CY, S-FH, and H-JL analyzed the literature and provided suggestions. W-CY, S-FH, and L-JH provided ideas and wrote the article. All the authors have approved the manuscript for submission.

## Conflict of Interest

The authors declare that the research was conducted in the absence of any commercial or financial relationships that could be construed as a potential conflict of interest.
